# Pulse oximeter provision and training of non-physician anesthetists in Zambia: a qualitative study exploring perioperative care after training

**DOI:** 10.1186/s12913-022-08698-5

**Published:** 2022-11-23

**Authors:** Meagan E. Peterson, Aviva S. Mattingly, Sylvia Bereknyei Merrell, Betelehem M. Asnake, Imraan Ahmed, Thomas G. Weiser

**Affiliations:** 1 Stanford University School of Medicine, 291 Campus Drive, 94305 Stanford, CA USA; 2 Department of Surgery, Massachusetts General Hospital, Boston, MA USA; 3 Department of Pediatrics, Stanford University, Stanford, CA USA; 4 Department of Anesthesia and Perioperative Medicine, UCLA, Los Angeles, CA USA; 5 Department of Anesthesia and Critical Care, University Teaching Hospitals, Lusaka, Zambia; 6 Department of Surgery, Stanford University, Stanford, CA USA

**Keywords:** Zambia, Anesthesia, Pulse oximetry, Capacity building

## Abstract

**Background:**

Pulse oximetry monitoring is included in the WHO Safe Surgery Checklist and recognized as an essential perioperative safety monitoring device. However, many low resource countries do not have adequate numbers of pulse oximeters available or healthcare workers trained in their use. Lifebox, a nonprofit organization focused on improving anesthetic and surgical safety, has procured and distributed pulse oximeters and relevant educational training in over 100 countries. We aimed to understand qualitatively how pulse oximetry provision and training affected a group of Zambian non-physician anesthetists’ perioperative care and what, if any, capacity gaps remain.

**Methods:**

We identified and approached non-physician anesthetists (NPAPs) in Zambia who attended a 2019 Lifebox pulse oximetry training course to participate in a semi-structured interview. Interviews were audio recorded and transcribed. Codes were iteratively derived; the codebook was tested for inter-rater reliability (pooled kappa > 0.70). Team-based thematic analysis identified emergent themes on pulse oximetry training and perioperative patient care.

**Results:**

Ten of the 35 attendees were interviewed. Two themes emerged concerning pulse oximetry provision and training in discussion with non-physician anesthetists about their experience after training: (1) Impact on Non-Physician Anesthetists and the Healthcare Team and (2) Impact on Perioperative Patient Monitoring. These broad themes were further explored through subthemes. Increased knowledge brought confidence in monitoring and facilitated quick interventions. NPAPs reported improved preoperative assessments and reaffirmed the necessity of having pulse oximetry intraoperatively. However, lack of device availability led to case delays or cancellations. A portable device travelling with the patient to the recovery ward was noted as a major improvement in postoperative care. Pulse oximeters also improved communication between nurses and NPAPs, giving NPAPs confidence in the recovery process. However, this was not always possible, as lack of pulse oximeters and ward staff unfamiliarity with oximetry was commonly reported. NPAPs expressed that wider pulse oximetry availability and training would be beneficial.

**Conclusion:**

Among a cohort of non-physician anesthetists in Zambia, the provision of pulse oximeters and training was perceived to improve patient care throughout the perioperative timeline. However, capacity and resource gaps remain in their practice settings, especially during transfers of care. NPAPs identified a number of areas where patient care and safety could be improved, including expanding access to pulse oximetry training and provision to ward and nursing staff to ensure the entire healthcare team is aware of the benefits and importance of its use.

## Background

Monitoring the surgical patient’s vital signs can allow for early recognition of surgical or anesthetic complications, which in turn, can lead to timely intervention. Oxygen saturation measured via a pulse oximeter is recognized as a key vital sign to monitor in order to detect hypoxemia earlier than clinical signs alone. Such monitoring is important to reduce perioperative morbidity and mortality. The first commercial pulse oximeter was introduced in the late-1970s and over the next two decades was incorporated into clinical care. One of the initial widespread uses was in anesthesia, where, in 1986, it was included in mandatory monitoring standards recommended by leaders in the field. [1, 2] Just a few years later in the late 1980s pulse oximetry had expanded to settings beyond the operating room and were recognized as a key monitoring device for early detection of hypoxemia. [3] Now, pulse oximetry is a mainstay of patient care and measuring oxygen saturation during vital signs assessment in high-income countries (HICs) is routine.

Although its importance in patient monitoring has been identified, many low- and middle-income countries (LMICs) still lack adequate access to pulse oximeters. [4] Efforts to change this have been initiated by increased dedication to equitable access to safe surgical care. WHO published the Guidelines on Safe Surgery in 2009, providing the first report to identify key aspects of safe surgical care. The Guideline recommends the use of pulse oximetry monitoring during surgery in LMICs as a means to detect hypoxemia and prevent airway and respiratory complications. However, the cost of and adequate training in the use of pulse oximeters was an important consideration. [5, 6] In addition to the WHO Guidelines on Safe Surgery, the 2015 Lancet Commission on Global Surgery united global public health support around the recognition of safe surgery as a right and bolstered commitments to increasing surgical capacity and perioperative training globally. [7]

Expansion of pulse oximetry use and training is one targeted effort that can improve patient care and safety in the perioperative space. While it is known that pulse oximetry education provides immediate improvement in knowledge of its use through quantitative and descriptive studies, to our knowledge, no qualitative study has been conducted focusing on how pulse oximetry training and provision has affected the clinical practice of non-physician anesthetists (NPAPs) in LMICs. [4, 8–12] Therefore, we conducted semi-structured qualitative interviews with non-physician anesthetists in Zambia to understand qualitatively how pulse oximetry provision and training affected them and their perioperative care and what, if any, capacity gaps remain.

## Methods

### Setting

In Zambia, anesthesia providers come from multiple training backgrounds: Consultant anesthesiologists, who are physicians with further specialty training in anesthesia; nurses with additional training in anesthesia to become Nurse Anesthetists; and Clinical Officer Anesthetists, who are graduates of an advanced diploma in clinical anesthesia program. Nurse Anesthetists and Clinical Officer Anesthetists work throughout the country and can practice in a hospital with or without a consult physician anesthesiologist present. Throughout this manuscript, these two roles will collectively be referred to as non-physician anesthetists (NPAPs).

Lifebox is a non-profit organization aimed at promoting safer surgery and anesthesia around the world, particularly in Low and Middle Income Countries (LMICs), [13] and has distributed over 28,000 pulse oximeters designed specifically for use in low-resource settings in over 100 countries. Lifebox works closely with local healthcare workers to provide education about pulse oximetry use and the management of perioperative patients. Many courses are delivered each year, including in Zambia. The course in Zambia is open to anesthesia providers (physicians and non-physician anesthetists) from all hospitals and regions in the country and attracts providers representing multiple practice settings. It is delivered over four days by a team of instructors, including Zambian anesthesiologists, and covers Lifebox pulse oximetry training as well as Safer Anaesthesia From Education (SAFE) Obstetrics training. [14, 15] The training workshop includes lectures, hands on sessions, and pre- and post-training written exams on pulse oximetry material covered.

### Study Design

This qualitative study used semi-structured interviews and a descriptive qualitative inquiry approach.

### Recruitment

Thirty-five participants representing hospitals throughout the country participated in the 2019 Lifebox course, and all were invited to participate in this study through email and WhatsApp messages by the primary author (MEP). The initial study invitation was sent in July 2020 and two additional reminders were subsequently sent July–August 2020. There was no financial incentive to participate. Course participants were eligible for inclusion if they were a practicing non-physician anesthetist, participated in the Lifebox course in 2019, provided anesthetic care for at least six months prior to the Lifebox course, and had a working level of English for the interview. Interested participants first completed a demographic survey to indicate their willingness to participate and provide background characteristics and were subsequently scheduled for an interview. Recruitment stopped once thematic saturation was achieved in the interviews, which we estimated beforehand to be 10–15 participants since all participants underwent the same training. [16]

### Interviews

Semi-structured interviews were conducted in English via a Zoom or WhatsApp call by MEP from July–October 2020. The interview guide (Table 1) was developed by two authors (MEP and TGW) with expert feedback from an anesthesiologist (BMA). Audio recordings of the interviews were obtained after a verbal review of consent information by MEP and used to obtain deidentified transcripts through a professional transcription service. The transcriptions were reviewed by MEP for transcription errors prior to uploading in Dedoose for qualitative analysis (Dedoose Version 8.3.35, Los Angeles, CA: SocioCultural Research Consultants, LLC www.dedoose.com.).

**Table 1:** Interview guide questions

Interview Guide Questions
1. What was the most beneficial part of the training?
2. What was the least beneficial part of the training?
3. What important concepts were not covered in the training?
4. What concepts do you wish were covered more thoroughly?
5. What concepts were covered that you felt were unnecessary?
6. Was the training relevant to your clinical practice? Why or why not?
7. Do you think pulse oximetry monitoring changed surgical care for your patients? If so, how? If not, why not?
8. Has having a pulse oximeter changed your confidence in your ability to provide safe anesthesia care?
9. How did the training you received from Lifebox impact your confidence?
10. Would you be comfortable delivering anesthesia without a pulse oximeter? Why or why not?
11. You mentioned that X physicians/nurses could benefit from receiving a pulse oximeter and training. In your opinion, would the training need to be altered? If so, how? If not, why not?
12. You noted that a pulse oximeter was available X amount of the time? Tell me more about that.
13. Is there anything else that you would like to discuss about pulse oximetry training that we have not talked about?

### Reflexivity

All of the authors have experience working in global health research and participating in global health partnerships. The primary author (MEP) has a background in nursing and public health and is a current medical student, whose multidisciplinary background shaped the approach taken in conceptualizing study participants’ experiences. MEP’s medical and healthcare knowledge guided understanding participants’ perspectives. Attempts were made to take participants’ words at face value and the entire multidisciplinary research team was utilized to conceptualize the work. Field notes were taken after each interview and memos made throughout the analytic process by the primary author to reflect upon the process of data gathering and interpretation.

### Analysis

A codebook was developed inductively through an iterative process. Two transcripts were open coded using small segments of data by the primary author (MEP). The open codes were then combined into focused codes based on overlapping definitions and reapplied to the two initial transcripts. A third transcript was coded using these focused codes to further refine the initial codebook and apply the codes to new data. An inter-rater reliability (IRR) test was done with another author (ASM) based on the first three transcripts. The codebook was further iteratively adapted through discussion with several authors (MEP, ASM, TGW, SBM). Two additional transcripts were coded by the primary author, and IRR tests and codebook refinement were repeated until a final, stable codebook emerged and resulted in kappa ≥ 0.7 for all codes. [17] The final codebook was then applied to all transcripts by the primary author. Initial themes were developed by MEP and then multiple authors (MEP, ASM, TGW, SBM) met to discuss themes that were identified, which were then shared with all authors for feedback and review. [17, 18]

### Ethics

This study was approved by the Stanford University Institutional Review Board (IRB-56841). Written, signed informed consent was waived by the IRB. Verbal informed consent was obtained prior to the interview. This article adheres to the Standards for Reporting Qualitative Research (SRQR) guidelines. [19] All methods were carried out in accordance with relevant guidelines and regulations along with ethical approval and informed consent to participation.

## Results

**Fig. 1:**
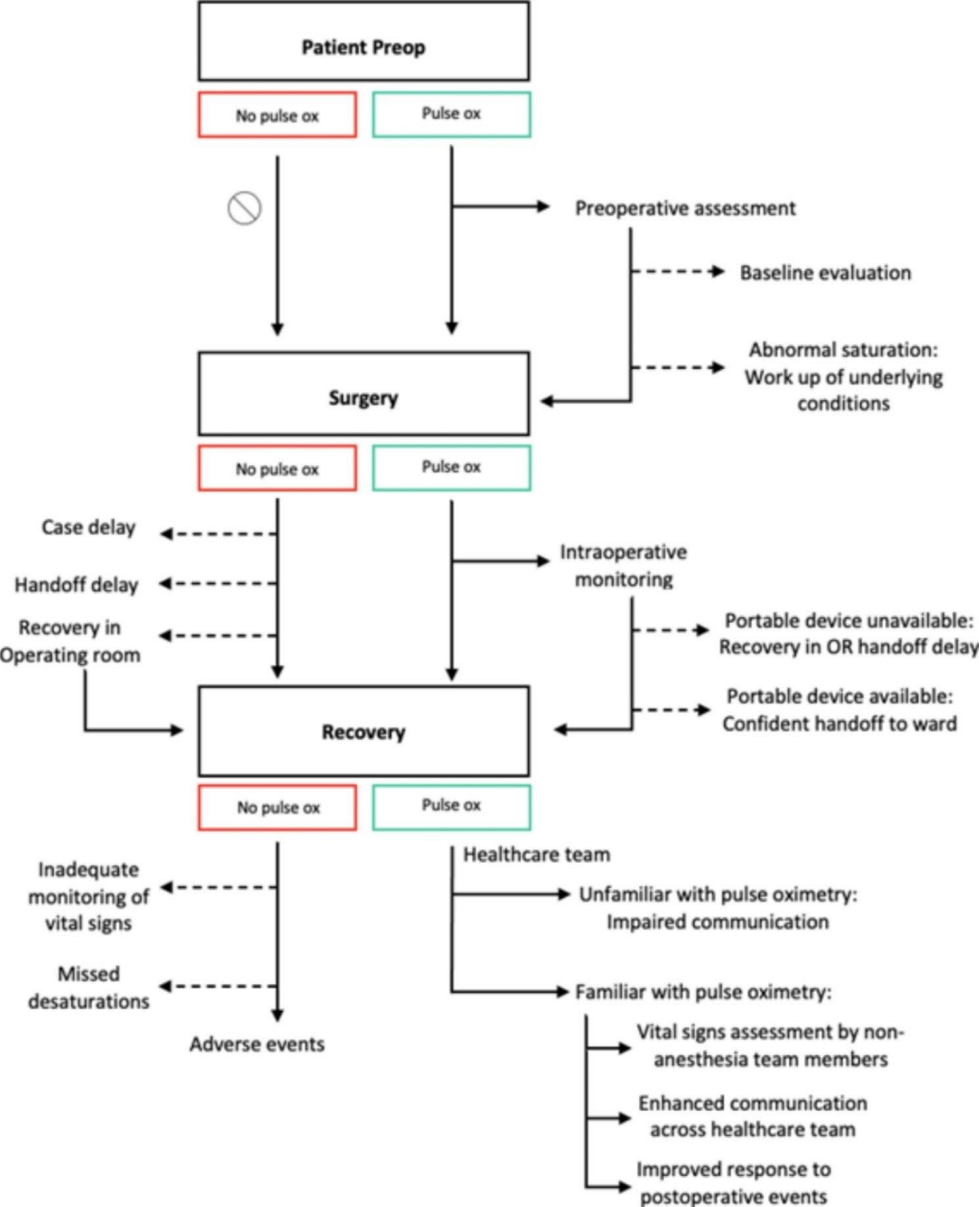
Perioperative Process Impacts. Pulse ox: pulse oximetry

We interviewed 10 of the eligible 35 course participants representing nine hospitals throughout Zambia (Table 2). Interviews ranged from 18 to 46 min in length and were 27 min on average. Two themes emerged concerning pulse oximetry provision and training in discussion with NPAPs about their experience: (1) Impact on Non-Physician Anesthetists and the Healthcare Team (Table 3) and (2) Impact on Perioperative Patient Monitoring (Table 4; Fig. 1). These broad themes are further explored through subthemes.

**Table 2:** Participant characteristics

Characteristic	Number of Participants, n = 10
Education	Nurse anesthetist, 5 Advanced diploma in clinical anesthesia (non-physician Clinical Officer Anesthetist), 4 Form V (equivalent to today’s Grade 12 General Certificate of Education), 1
Years of anesthesia experience	0–4 years, 8 5–9 years, 1 10–14 years, 1
Hospital characteristics where participants work (more than one may apply)	District, 6 Government, 5 Referral, 3 Rural, 2 Teaching, 2 Mission, 1 Urban, 1
Types of surgical cases most frequently providing anesthesia for (more than one may apply)	Obstetrics/gynecology, 10 General surgery, 9 Pediatric surgery, 7 Orthopedics, 3 Plastic surgery, 3 Urology, 2 Maxillofacial/otolaryngology, 1 Ophthalmology, 1
Approximate case load in last 2 weeks	10–14, 1 15–19, 3 20–24, 3 25–29, 1 40–45, 1 No response, 1
Of cases in last 2 weeks, approximate percent that were general anesthesia	0–24%, 5 25–49%, 2 50–74%, 2 75–100%, 1

**Table 3:** Theme 1: Impact on non-physician anesthetists and the healthcare team

Impacts	Representative Quotes
Knowledge and Confidence	“knowledge is power and I’m able to say this because of that workshop or that seminar which I went… Now if I didn’t have that knowledge I would have just said the patient saturation was this and this, without any interpretation, without any worry” “before … the Lifebox course, my knowledge on the pulse oximetry usage, oximeter usage was very limited. But when I did the course, I was given broad knowledge on the pulse oximetry. So it was quite beautiful for me” “But now, after going for that training, I have confidence in, on how to use [the pulse oximeter].” “you cannot talk about a pulse oximeter without talking about the patient… you discover that you are, you are learning more about…care of all these patients who are under anesthesia or post-operatively. … So, it’s some kind of reminds you of some, some concepts which you, you might have overlooked or forgotten for some time.” “I became more confident in managing patients under anesthesia, everyday monitoring them using the pulse oximeter, because we were able to share experiences”
Quality of Care	“After the training and then were given a machine to use, I think our patients are benefiting from that…. we are able to monitor patients the way they are supposed to be monitored.” “you cannot guarantee the safety of the patient without a pulse oximeter” “[intraoperative use of a pulse oximeter is] the standard that has to be followed so there is no compromise about it” “A pulse oximeter I think for me puts me very close to the patient. I know what is happening to the patient and then intervention can be done immediately there is a change.” “the moment you connect a, a pulse oximeter, it will make you to intervene more. And then hence, it has saving a life there.”
Workflow	“pulse oximeter is like this…it has something which has empowered me now, to move with it, and also wherever I go out and see the patient I have it, it has helped me so much” “Because I understand the magnitude and the importance of having the pulse oximeter around. I make sure I have it with me every time maybe I’m assessing patients pre-op for surgery.” “And when the volume is just there and you are near, you are able to hear ting, ting, ting, you appreciate that, ‘Oh, my patient is okay,’ because the sound is, correlates with the pulse.”
Healthcare Team Communication	“I’ll ask them, yes, how is the saturation? How is the pulse, how is the patients, you know, that will give me an idea what’s happening with a patient even if I’m not there. All because of the pulse oximeter.” “And there is a way it changes when it reaches 93, it will change 90, eighty-what, you know. Even as you go there, you know how to, to move, whether to rush there or to walk there.” “Also know when you have that tool, you are able to also educate others, how to use it, how to prepare the patient with it and all those things… sometimes …you find somebody has removed it, then you have to educate them no, this has to be there. When it is 100 then it is okay, 95 is okay when it just go down, you let me know I will come fast.”

**Table 4:** Theme 2: Impact on perioperative patient monitoring

Stage of Patient Care	Benefits	Gaps	Representative Quotes
Preoperative Assessment	Pulse oximetry 1) provides comprehensive baseline of oxygen saturation and pulse 2) prompts assessment of underlying conditions	Lack of pulse oximetry availability on the wards limits ability to perform preoperative assessment	“In this COVID era actually, we realize [the patient] had bilateral desaturation … he was COVID positive. But everything else was okay until we connected the pulse oximeter.”
Intraoperative Monitoring	Pulse oximetry allows for oxygen saturation and heart rate monitoring during surgery Lifebox training 1) incorporates use of Surgical Safety Checklist 2) reiterates key anesthesia principles e.g. treatment of hypoxia, laryngospasm etc.	Lack of pulse oximetry in all operating rooms delays cases	“I had a case that it was just a cesarean section. … I didn’t have the pulse oximeter, I looked around. … I wouldn’t start a case without a pulse oximeter.”
Postoperative Assessment	Pulse oximetry 1) allows objective assessment by non-anesthesia providers 2) improves communication between the PACU/ward team and anesthesia team 3) Allows recognition and treatment of hypoxia in the immediate postoperative period	Lack of pulse oximetry outside of the operating room delays transfer to recovery area Some recovery areas have no resources to monitor patients and/or nurses do not have training Some hospitals do not have designated recovery areas and patients are transferred from the operating room to wards that do not have monitoring capabilities	“being trained, going to school doing anesthesia … makes it difficult for me to just do short cuts and pretend all is well… because of lack of a pulse oximeter in my recovery, I’m forced to keep my patients on the table until they recover.” “Before I had the pulse oximeter, I wasn’t so confident leaving my patients in the post-operative ward… but now I can confidently do that because I can see the readings.”
ICU and Ward Care	When monitors are not available in wards, a portable pulse oximeter 1) allows anesthesia to assess saturation in the ICU 2) facilitates work up of respiratory conditions	Lack of pulse oximetry on the wards requires anesthesia providers to borrow operating room pulse oximeters when evaluating critically ill patients Nurses on all wards are not familiar with the pulse oximeter	“when we’re going out there to see patients, in ICU … I have to literally go back to theatre and get the one I use … For a short time and take it back.” “the patient can be lying on the table, on the bed without realizing that the saturation is going down. If those nurses - even other people in the theatre - can have that knowledge they will be quick to act.”

### Impact on non-physician anesthetists and the Healthcare Team

#### Knowledge and clinical decision making

NPAPs described how having pulse oximetry training helped boost confidence in their clinical care. For some, this was due to directly learning new information about how a pulse oximeter worked and how to interpret the findings, while for others it served as a reminder of core monitoring tenets. They reported that their increased knowledge led to better interpretation of oximetry values when assessing a patient. Confidence was also built through sharing experiences during the course with anesthesia providers from around the country.

#### Patient safety

Pulse oximetry monitoring is consistent with NPAPs’ values of patient safety and high-quality care. Providers highlighted that using pulse oximetry allowed them to monitor patients appropriately and was a standard that should not be compromised, especially intraoperatively. They felt that pulse oximetry contributed considerably to patient safety by alerting them immediately to changes in patient status, allowing for life saving quick interventions.

However, knowing the right intervention in the case of patient decompensation did not always equate to a sense of improved quality when resources were lacking, which created internal tension as described by one provider, “So you discover that you are almost the only one who is fully knowledgeable…So you just be there and watch. But your heart tells you, we can do better than this, but resources are somehow holding you back.” Multiple NPAPs discussed how they are doing their best to adapt in their environment but find it difficult to monitor at the level of care they would like to provide due to resource and capacity constraints.

#### Workflow

NPAPs felt that having a portable device further enhanced their ability to provide quality care in line with their values. Many NPAPs began carrying the pulse oximeters with them at all times when they went to see patients. This led to multiple NPAPs describing taking additional steps to ensure they had a pulse oximeter in critical moments, such as ensuring extra batteries were always on hand to power the device in case of a power outage or going to another operating room to find a pulse oximeter for their case.

NPAPs appreciated how the audible noise from the pulse oximeter integrated into their daily clinical care. For example, many of the NPAPs relied on the audible sound of the pulse oximeter to reassure them of patient status during the perioperative periods, such as when drawing up medications or when monitoring patients “even from afar”, as is often necessary given the setup of postoperative recovery areas and the lack of enough monitors.

However, two NPAPs mentioned the strain on their workflow and ability to monitor patients appropriately because they do not have ready access to a portable pulse oximeter. One NPAP mentioned that the portable pulse oximeter in their facility is kept under lock and key and difficult to access, which impairs their ability to do pre- and postoperative assessments on the wards. The other lacked a portable pulse oximeter in their facility and, therefore, keeps their patients in the operating room to recover rather than in a recovery area.

#### Healthcare Team

Improved perioperative monitoring impacted healthcare team communication, provided teaching moments, and furthered capacity building. Adequate pulse oximetry monitoring improved communication within the healthcare team by providing objective data about patient status that could be communicated from ward nurses to the anesthetists.

However, NPAPs frequently mentioned that few ward nurses had pulse oximetry training and experience. One NPAP mentioned that in their hospital “when you say pulse oximeter, the language is a bit alien to [many nurses],” which is having a negative impact on staff and patient care. Another NPAP described several instances where it was difficult to recover a patient appropriately because only the anesthesia team had pulse oximetry education and further advocated for additional training of other members of the healthcare team.

### Impact on Perioperative Patient Monitoring

#### Preoperative

NPAPs valued the portable pulse oximeter as a means to better understand the importance of a preoperative assessment. The addition of a more thorough preoperative assessment and screening process allowed providers to feel more confident in developing an appropriate anesthesia plan. The portability of the Lifebox pulse oximeters allowed for patient assessment on wards, which generally lack pulse oximetry monitoring capabilities. One NPAP reported that temperature and blood pressure were routinely documented on the wards, but only with a portable pulse oximeter was it possible to record oxygen saturation and pulse, which provided a better baseline for surgical patients.

The ability to conduct a more thorough pre-assessment led to catching unknown diagnoses, such as congenital heart disease and COVID-19, and appropriate follow up investigations prior to the scheduled operation. Several NPAPs mentioned having a pulse oximeter allowed them to detect hypoxemia even when the patient appeared clinically well.

#### Intraoperative

The NPAPs interviewed reported the ability to monitor oxygen saturation using monitors or anesthesia machines in operating rooms prior to the Lifebox course. A specific intraoperative improvement included integrating knowledge of pulse oximetry and the Safe Surgical Checklist by one NPAP and, more generally, the overall benefit of being able to closely monitor patient status.

#### Postoperative

Several NPAPs reported considerable changes in the postoperative patient monitoring after receiving a pulse oximeter and having additional training. Multiple stated how they feel more confident because pulse oximetry provides a way to monitor the patient until they are “out of danger” and recovered from anesthesia. Another described the benefit of being able to identify concerns early based on declining oxygen saturation, which allowed for appropriate interventions. More widely available postoperative monitoring was a welcomed change in practice.

However, beyond the pulse oximeter, many NPAPs described facilities with insufficient supplies and monitoring capacity. One NPAP stated that the recovery area “doesn’t even have anything. It only has beds… So, no monitors, no oxygen concentrators, no cylinders, no suction machines.” In this situation, having a pulse oximeter was beneficial because they could at least have “the basic vitals”.

The lack of postoperative monitoring left some NPAPs concerned about patient safety outside the operating room since the “ward which is receiving the patient does not have even a patient monitor or a pulse oximeter”. At times, the lack of postoperative monitoring capacity was due to NPAP time constraints, and other times it was due to resource limitations. One NPAP shared a story of patient losses in situations where there were no pulse oximeters or monitors in the postoperative recovery area and patient decompensation was not recognized. However, they went on to describe an alternative scenario of a pulse oximeter being used, which would have provided an early warning of a complication, quick intervention, and “would have saved a life”.

#### General wards and intensive care units (ICUs)

NPAPs also saw utility in using pulse oximetry outside of perioperative care and reported benefit on general wards as well, such as for assessing respiratory patients in an area with a high incidence of pneumonia. Access to pulse oximetry “has changed the way [anesthetists] monitor patients who are in critical conditions” beyond the scope of the operating room. NPAPs suggest benefits for non-surgical patients throughout the hospital when there are enough pulse oximeters available. Being given a portable pulse oximeter allowed anesthetists to monitor patients on the wards and in the emergency room, instead of only having a fixed machine in the operating room for monitoring. However, many NPAPs spoke of having no pulse oximeter in wards or ICUs “unless [they] go with it”.

## Discussion

Through qualitative interviews, we assessed how pulse oximetry training and provision affected a cohort of non-physician anesthetists and their perioperative care in Zambia. We found that NPAPs were more confident in their ability to provide safe perioperative care with additional training and expanded monitoring capabilities via pulse oximetry. We also found inter-team communication improved when pulse oximeters were available in postoperative recovery areas so anesthetists could better understand patient status as reported by bedside nurses. Together, these led to qualitative improvements in care for surgical patients. However, continued barriers to perioperative patient care were found in settings lacking dedicated postoperative recovery areas or post-anesthesia care units (PACUs), lacking the ability to monitor oxygen saturations regularly on the wards, and nurse unfamiliarity with pulse oximetry.

Previous studies have explored healthcare provider confidence and the impact of and barriers to pulse oximetry use in LMICs, but, to our knowledge, this is the first qualitative study focusing on these aspects in non-physician anesthetists in Sub-Saharan Africa. Our study found perceptions among NPAPs of improved self-reported confidence and knowledge after training, improved monitoring capacity, and sharing experiences with other anesthesia providers. Perceived improved confidence in the clinical decision making of clinical officers and nurses after pulse oximetry and specialty-specific training has also been seen in Malawi among healthcare workers caring for children with suspected pneumonia.[20] However, education alone may not be enough to improve confidence and a thoughtful approach to setting and context and clinical role within the healthcare team need to be considered for future education capacity building initiatives.[21].

Surgical and anesthesia capacity continues to be constrained in Zambia and targeted improvements in this area could improve perioperative care. [22–24] Qualitative studies in other Sub-Saharan African countries have also found that while many providers acknowledged pulse oximetry assessment was important for hospitalized patients, its use was limited by inadequate availability of pulse oximeters and inadequate education on oximetry benefits for the entire healthcare team. [25–27] Our study found that participant non-physician anesthetists perceived that, often, nurses did not have training in pulse oximetry monitoring and some hospitals lacked pulse oximetry in wards and postoperative recovery areas. Further expansion of pulse oximetry training and monitoring capabilities, especially for staff caring for surgical patients, represents one targeted area where healthcare delivery in these settings in Zambia could be improved.

Postoperative mortality is higher in Zambia and other LMICs compared to HICs and remains an area where increased capacity building could substantially decrease surgical morbidity and mortality. [28–31] When designing interventions to strengthen surgical care in LMICs, the entire continuum of the surgical patient’s stay in hospital should be considered. Although developing equipped PACUs de novo is time and resource intensive, once instituted, it can have tangible patient care benefit. [32] Our study revealed that, from the perspective of a cohort of non-physician anesthetists, patient safety could be improved through continued strengthening of postoperative recovery areas that include additional resources and monitoring capabilities, even beyond pulse oximetry.

Future surgical capacity building initiatives should be interdisciplinary in nature and inclusive of physicians, non-physician clinicians, and nurses. Our study found that NPAP participants perceived that nurses, who had not undergone the pulse oximetry training with participants, would benefit from the knowledge and skills participants gained in patient monitoring through pulse oximetry. Nurses can play a key role in early detection of patient complications and should be included in global capacity building initiatives aimed at improving care for surgical patients. In Zambia, there is an ongoing educational initiative to train nurses in the topics and skills that are essential to working in a critical care ward as they strive to provide care for increasingly complex medical and surgical patients. [33] Additionally, a recent priority-setting study highlights the desire of perioperative nurses within Africa to continue prioritizing translating research into their practice and implementing safety procedures into their practice such as utilization of the safe surgery checklist and infection control principles. [34] Keeping the entire perioperative care team, as well as the entire perioperative period, in mind when designing education initiatives would greatly benefit perioperative patient care.

For years, many have advocated for the expansion of pulse oximetry provision and education, especially in the perioperative space. However, the COVID-19 pandemic has further demonstrated the need for early detection of hypoxemia as a key vital sign for all patients and highlighted the inequitable distribution of pulse oximeters and pulse oximetry training. [35, 36] Despite organizations, such as Lifebox, distributing thousands of pulse oximeters in LMICs throughout the pandemic, there continues to be need. Efforts should continue after the pandemic for sustainability and continued improvement in patient monitoring capabilities. [13]

This study is not without its limitations. Due to the COVID-19 pandemic, audio-only interviews were conducted instead of in-person interviews. Audio-only interviews are an accepted method for qualitative semi-structured interviews, but it meant we could not participate in additional observations to supplement the interviews. [37] Such observations may have allowed for a more comprehensive understanding and analysis of workflow and structural barriers to perioperative pulse oximeter use by directly observing how participants use pulse oximetry within their hospital setting. Although study participants worked in a variety of settings throughout Zambia, their experience may not be representative of all perioperative settings in the country. Additionally, participants may have been hesitant to provide negative information about Lifebox or pulse oximetry. Steps were taken to mitigate this concern, such as Lifebox not having access to study participant names and the interviewer not being a Lifebox affiliate. Limitations notwithstanding, we believe this work will be beneficial for organizations and individuals working in global surgery and capacity development. While the exact successes and difficulties in introducing pulse oximeters and pulse oximetry training will differ depending on the context, since each healthcare system and workforce will have different educational needs and potential structural barriers, this study will provide readers important programmatic considerations.

## Conclusion

Non-physician anesthetists reported tangible and intangible benefits to attending a Lifebox training course and using pulse oximetry for perioperative monitoring. They described having increased confidence and assurance knowing they would be able to intervene quickly and appropriately in case of an emergency. However, most NPAPs also described a continued tension between the monitoring standards they consistently aim to provide and actual practice possible in a constrained environment due to a lack of resources and lack of other healthcare workers, most commonly ward nurses, trained in pulse oximetry. This study demonstrates the positive impact that capacity building courses can have on non-physician anesthetists and patient care and highlights important points of consideration that must be made for sustainable change and improvements to occur. Continued work is needed to meet global surgery goals.

## Data Availability

The dataset supporting the conclusion of this article are available from the corresponding author on reasonable request.

## List of abbreviations

HICs: High-income countries
ICUs: Intensive care units
LMICs: Low- and middle-income countries
NPAPs: Non-physician anesthesia providers, also referred to as non-physician anesthetists
PACUs: post-anesthesia care units
SAFE: Safer Anaesthesia From Education
SRQR: Standards for Reporting Qualitative Research
